# Doubled-Edged Swords in the Biology of Conflict

**DOI:** 10.3389/fpsyg.2018.02625

**Published:** 2018-12-20

**Authors:** Robert M. Sapolsky

**Affiliations:** ^1^Gilbert Laboratory MC 5020, Departments of Biology, Neurology and Neurological Sciences, and Neurosurgery, Stanford University, Stanford, CA, United States; ^2^Institute of Primate Research, National Museums of Kenya, Nairobi, Kenya

**Keywords:** amygdala, frontal cortex, testosterone, oxytocin, violence

## Abstract

Considerable advances have been made in understanding the biological roots of conflict, and such understanding requires a multidisciplinary approach, recognizing the relevance of neurobiological, endocrine, genetic, developmental, and evolutionary perspectives. With these insights comes the first hints of biological interventions that may mitigate violence. However, such interventions are typically double-edged swords, with the potential to foster conflict rather than lessen it. This review constitutes a cautionary note of being careful of what one wishes for.

## Introduction

We humans are animals—mammals, Old World primates, apes; in other words, we are nothing more or less than biological organisms, and everything we do must be viewed in the context of our biology. This is a truism to anyone in the life sciences, but often seems to border on hegemony to those in the social sciences. This latter view is due to too narrow of a read of what “biology” means in the context of human behavior (a narrowness, it should be noted, propagated by many a life scientist). Little will be understood about humans if scientists proclaim the identification of ***the*** brain region, or ***the*** gene, or ***the*** hormone or neurotransmitter that supposedly explains everything. Instead, in order to understand why a human social behavior has occurred, one must factor in neurobiological events 1 s before, but also endocrine events from days before, neuroplasticity from weeks before, epigenetic events in childhood, fetal environment, the genome in a fertilized egg, culture, ecology, and evolution. We cannot understand human behavior outside this broad context of biology; at the same time, that very broadness means that such biology is intimately and symmetrically intertwined with the traditional domains of social science.

Research concerning the underpinnings of human social behavior has expanded enormously among neuroscientists, endocrinologists, geneticists, evolutionary biologists, and so on. As one measure of this expanding interest, the annual meeting of the Society for Neuroscience consistently attracts more than 30,000 researchers. A growing focus of such research has been on the biology of conflict, and much has been found out. And not for the first time, with the increase in understanding of a problem comes the desire to intervene and to correct. This can work wonders in areas ranging from vaccine development and surgery on fetuses to the fact that there are people who know how to fix cars. But the study of the biology of human behavior is no less vulnerable to fads and bandwagons than many other ventures, and they have certainly emerged with respect to the biology of conflict. This subject is rife with the law of unintended consequences, where one should, in effect, be careful of what is wished for. This non-technical review considers some realms where seemingly straightforward biological features of human conflict are anything but straightforward, producing a number of double-edged swords for those who contemplate interventions. The tone throughout, in touching on some of the more notable neurobiological domains that are filled with promise, will be one of caution, where an overabundance of enthusiasm may be unwarranted.

## The Promise of Neuroplasticity

The first years of life are obviously critical to the sort of brains we possess as adults; however, such importance can readily lead to the incorrect conclusion that the trajectory of the brain is set in stone early in life (Bruer, 2002). Instead, at all stages of life, the brain can change in response to experience. Few domains of neuroscience have so fueled excitement, among scientists, and non-scientists alike, than the promise of such “neuroplasticity” (Doidge, 2007).

Neuroplasticity in the brain plays out on a variety of levels. The most fundamental version has been recognized for half a century, and concerns synapses, the connections by which one neuron communicates with another. Excitation in one neuron (an “action potential”) is not guaranteed to similarly excite the next neuron in line, and repeated stimulation of a particular synapse leads it to be “strengthened,” which is to say that excitation in one neuron is now more likely to be propagated to the next in line. This potentiation of such pre-existing synapses has long been viewed as one of the fundamental ways by which learning occurs.

Neuroplasticity occurs on a larger scale as well. New synapses will form and others retract in response to experience. Axons and dendrites, the cables by which neurons reach out to other neurons and form synapses with them, can sprout new branches and retract old ones. If someone becomes a serious musician, sprouting occurs such that the amount of space devoted in the auditory cortex to the sound of his instrument expands (Pantev and Herholz, 2011); if a volunteer is blindfolded for days, projection fields into the cortex expand so that the acuity of other senses increases as a compensation (Merabet and Pascual-Leone, 2010). As one of the biggest revolutions in decades in neuroscience, dogma has been overturned with the demonstration that the adult brain can make new neurons (Kempermann et al., 2018) (although the extent of it in the human brain has been questioned recently Sorrells et al., 2018). Moreover, such “adult neurogenesis” occurs in response to stimulation, exercise, and as reparative compensation in the injured brain. As a result of changes in neuron number, synapse number and complexity of circuitry, entire regions of the brain can change in size. Become a London cab driver, which demands forming a complex spatial map of every street and building in the city, and the part of the brain most responsible for this, the hippocampus, enlarges (Maguire et al., 2000). If a monkey is moved into a larger social group than its current one, his frontal cortex will grow larger, particularly so if he rises high in the dominance hierarchy (Sallet et al., 2011).

The potential for neuroplasticity readily fuels enormous optimism—with informed harnessing of these processes, everything is possible, from coaxing severed spines into reconnecting to turning the neural circuitry of hate into love (see Cortright, 2015, for an example of particularly unbridled enthusiasm in this regard). And here is where double-edged swords come in. As an example within a narrow neurological framework, new neurons will be born in the hippocampus after a hypoxic injury, certainly a good thing in principle; however, a newly-born neuron attempting to integrate into the pre-exiting circuits of an adult brain is a different proposition than in a fetal brain, and mis-wiring in the former can produce the self-stimulating loops that generate seizures (Parent et al., 2006).

The double-edge to neuroplasticity is clear when it comes to the behavioral concerns of this review. Neural plasticity allows the hippocampus to enlarge, enhancing spatial memory skills. But at the same time, the same neurobiology allows the amygdala, a brain region central to fear, to enlarge and contribute to the crippling state that constitutes post-traumatic stress disorder. Similarly, the same neural plasticity that underlies the conversion of a Them to an Us, mediating reconciliation and forgiveness, makes possible the conversion of an Us to a Them. The plasticity that allows individuation and perspective-taking to begin to elicit empathy for a Them is similar to how stress and fear can blunt the neural capacity for empathy. After all, physiologically, the opposite of love is indifference rather than hate, and learning to love and learning to hate are both contingent on the neurobiology of learning. In other words, neuroplasticity is value-free, and a new or newly strengthened synapse can be for the better or worse.

## The Promise of Empathic Mirror Neurons

Both neuroscientists and the general public have been fascinated by “mirror neurons.” The core findings concerning them are far from the focus of this paper: when we are intent on performing a particular movement, Movement X, a particular array of neurons in the “pre-motor cortex” activate in anticipation of the movement, soon followed by activation of a cognate array of neurons in the “motor cortex,” which then commands muscles to actually carry out the behavior. As first described in the 1990s, about 10% of the pre-motor neurons that would activate when one is about to make Movement X activate as well when observing someone else doing Movement X (Rizzolatti et al., 1996).

The activity of these “mirror neurons” can be remarkably sophisticated. For example, some neurons that cause Movement X will activate as mirror neurons when an individual is merely hearing the sound of someone else doing Movement X. Or consider Movement X consisting of lifting a tea cup from a table; different subsets of Movement X neurons will serve as mirror neurons when observing the cup being lifted in order to drink from it, vs. being lifted to clear the table (Rizzolatti and Craighero, 2004). Thus, mirror neurons can encode intent. When we watch a performer on a tightrope and unconsciously stick our arms out to stabilize our own balance, this occurs because mirror neuron activity in the pre-motor cortex was strong enough to activate neurons in the motor cortex, producing actual behavior.

Most in the field agree that mirror neurons are central to observational learning. This is both to mimic a behavior, as in a choreographer demonstrating a movement for the dancers, and as in learning that, for example, an object should not be touched by watching someone else leap back in pain after having done so (Molenberghs et al., 2009). Feverish debate has emerged with speculation that mirror-like neurons exist in other cortical regions, and are responsible for understanding what someone else is thinking, and for being capable of taking their perspective (Gallese and Goldman, 1998). Even more controversial has been the speculation that such neurons are also responsible for us understanding what someone else is feeling—the neurobiological substrate of feeling someone else's pain (Kaplan and Iacoboni, 2006) [and the speculation that mirror neurons aid understanding other people's thoughts and feelings has prompted the “broken mirror” theory of autism (Hamilton, 2013)].

The possibility that mirror neurons mediate empathy has caused tremendous excitement, including among neuroscientists [one of whom, an eminent figure in the field, has stated “Mirror neurons will do for psychology what DNA did for biology,” and has even called these cells “Gandhi neurons” (Ramachandran, 2000)]. This suggests a prescription of more mirror neuron-ing in our empathy-starved world. However, there is a double-edged sword in a prescription of enhancing mirror neuron-mediated empathy; as an example that dominated American news in recent months, one can feel empathy for a woman whose claim of having been assaulted you believe, or feel empathy for a man whose claim of being falsely accused you believe.

More importantly, the biggest problem with mirror neurons and empathy is in the use of the word “putative” in the previous sentence. In the quarter century since mirror neurons were first described, speculation about their role in empathy continues. However, to date, few to no data exist that actually implicate them in this role. In the words of one respected psychologist, mirror neurons are, “the most oversold idea in psychology” (Hickok, 2014).

## The Promise of Administering Oxytocin Far and Wide

Brains obviously do not function as islands, and instead are constantly influenced by sensory stimuli, nutritional status, bodily states, and so on. Among the most powerful modulators of brain function are hormones. Particular hormones are secreted from particular glands (e.g., estrogen from the ovaries), circulate in the bloodstream, and alter the function of cells throughout the body; of great importance, cells altered by hormones includes neurons in the brain. For example, the epinephrine that is secreted from the adrenals if one were being chased by a lion, not only promotes the delivery of energy from the liver to thigh muscles, but also causes the brain to focus and concentrate.

Few hormones are as interesting to a review such as this as is oxytocin. The hormone, secreted from the pituitary gland, is responsible for mother-infant bonding among mammals, an evolutionary development of obvious adaptive advantage. Moreover, in the relatively few mammalian species that show stable monogamy, oxytocin mediates the formation and maintenance of pair bonds (Donaldson and Young, 2008). As a recent finding, oxytocin is secreted by both dogs and their owners in response to them making eye contact, and boosting oxytocin levels prolongs the eye contact (Nagasawa et al., 2015); this is remarkable, in that this hormone, which has been promoting mother-infant bonding for 60 million years has, in the last few tens of thousands of years of dog domestication, been co-opted for this novel role.

Most importantly, oxytocin promotes pro-social behavior in a variety of ways. In both laboratory experiments and naturalistic settings, it makes people more trusting, forgiving, empathic and charitable. It improves the accuracy of reading people's emotions. Moreover, oxytocin makes people more responsive to social cues and social feedback (Meyer-Lindenberg et al., 2011).

These pro-social effects prompt easy speculations about the benefits of administering oxytocin to humans. Which is where this hormone's double-edged sword emerges. Excellent recent work has shown that oxytocin does indeed promote pro-social behavior, but crucially, only toward in-group members. In contrast, when dealing with out-group members or strangers, oxytocin's effects are the opposite. In such settings, the hormone decreases trust, and enhances envy and gloating for the successes and failures, respectively, of the out-group member. Moreover, the hormone makes people more pre-emptively aggressive to out-group members, and enhances unconscious biases toward them (De Dreu et al., 2011a,b; De Dreu, 2012). In other words, a hormone touted for its capacity to enhance pro-sociality does no such thing. Instead, what it does is worsen Us/Them dichotomies, enhancing in-group parochialism as well as out-group xenophobia. This is certainly not the hormone to cure our ills.

## The Promises of Banishing Testosterone and, as Long as We're at It, Males

A revisionist picture also applies to testosterone, the biological factor probably most immediately associated with violence – after all, in the vast majority of social species, and in every culture examined, males are more likely to be aggressive than females. Moreover, as something of a gold standard in endocrinology, subtraction of the hormone (i.e., through surgical or chemical castration) decreases average levels of aggression in humans and other mammals. Finally, if testosterone-like androgens are administered in high amounts (for example, as among athletes who are steroid abusers), levels of aggression typically rise.

The malign effects of testosterone run even deeper, if more subtly than that. The hormone decreases trust and biases people to perceive neutral faces or circumstances as threatening, thus lowering the threshold for provocation. Empathy is inhibited as well, and the accuracy of Theory of Mind about other people's thinking declines. Testosterone also biases toward risk-taking, makes individuals less sensitive to the typically constraining effects of negative reinforcement, and less cooperative because of an exaggerated sense of the importance and accuracy of one's thinking (Hermans et al., 2006; Bos et al., 2010, 2012). None of these effects auger well for reducing interpersonal and intergroup conflict.

Yet, there is much reason to doubt the importance of testosterone to violence. To begin, testosterone boosts spontaneous aggression only when levels are pushed into the “pharmacological” range (i.e., higher levels than the body normally ever generates); within the normal range, individual differences in testosterone levels do not predict subsequent levels of aggression (as measured in a variety of species, with a variety of endpoints) (Archer, 2006). Furthermore, testosterone does not “cause” aggression. Instead, it amplifies pre-existing social tendencies toward aggression, increasing the intensity of amygdaloid excitation once the region is activated, and lowering the threshold for such activation (Kendrick and Drewett, 1979). As a behavioral manifestation of this, if a middle-ranking primate is administered high levels of testosterone, his levels of aggression typically rise. However, this does not involve his now threatening higher-ranking individuals; instead, he simply displaces aggression more often on lower-ranking ones (Dixson and Herbert, 1977).

Therefore, testosterone amplifies, rather than causes aggression. Recent work has revealed an even subtler, more interesting view of the hormone. As originally proposed as the “challenge hypothesis,” testosterone does not even amplify pre-existing social tendencies toward aggression. Instead, it amplifies pre-existing social tendencies toward whatever behaviors are needed to maintain status when it is being challenged (Wingfield et al., 1990). For most social mammals, this distinction is irrelevant, in that aggression is the means by which a male maintains high status when challenged. In humans, however, there are many ways to maintain status. For example, consider auctions for a charitable cause, as bidders compete for alpha status of conspicuous largesse. This has been explored in some remarkable studies involving economic games where status is accrued through generous offers to other players, and by a reputation for being trustworthy. In such a setting, testosterone enhances generosity and other pro-social behaviors in players (Eisenegger et al., 2010; van Honk et al., 2012; Wibral et al., 2012). In other words, if we suffer from a surfeit of testosterone-mediated violence, a significant cause lies in the fact that we reward violence with status so readily.

The complexities concerning testosterone's actions segue into a consideration of the complexities of males in group. Within a troop of savanna baboons, there is typically high rates of escalated male-male aggression, something that dominates everyday life. In contrast, levels of male-male aggression within a group of chimpanzees are typically lower. The explanation lies in the differing life histories of baboons and chimpanzees. Among the former, males leave their natal troop around puberty, transferring into another troop; as a result, in any troop, the adult males are typically unrelated and grew up a scattering of other troops, with no histories of affiliative or cooperative behaviors with each other. In contrast, male chimpanzees spend their lives in their natal group, so that the adult males are often siblings or relatives, or at least individuals who have known each other since childhood.

This picture of chimpanzee males shows the benefits of male familiarity and cooperation. Naturally, there is a double-edged sword, in that chimpanzee males are capable of something that no baboon males in a troop could ever organize. Specifically, the male chimpanzees in a group carry out organized and coordinated “border-patrols,” and will attack and even kill any male encountered from a neighboring group (Wrangham and Peterson, 1996). As documented more than once, such killings can extend to the point of eradicating all the males in the adjacent group. A group of males getting along well can be a very frightening thing for the neighbors.

The phenomenon of related male chimpanzees cooperating raises an implicit double-edged sword about human cooperation. A cornerstone of thinking about the evolution of behavior is the importance of relatedness. Enhancing the number of copies of one's genes passed into the next generation is not only accomplished with individual selection and reproduction, but also “kin selection,” the aiding of relatives in doing the same. The extent of such cooperation should vary as a function of the degree of relatedness, summarized in the famous quip, “I'll gladly lay down my life for two brothers or eight cousins.”

A key prerequisite for kin selection is, of course, being able to recognize how related someone is to you. Among most mammals, this is accomplished through olfaction, where individuals carry pheromonal signatures that resemble those in other individuals as a function of the degree of relatedness. A rodent, for example, can distinguish between full-, half-siblings, cousins and strangers based on pheromones. In contrast, the human capacity for kin recognition through pheromones, or through any innate mechanism, is minimal, at best. Instead, humans have to think through who is a relative, relying on memory and reasoning. Herein lies one of the most defining things about human sociality, namely that we can *feel* more related to someone than we actually are. This capacity for “pseudo-kinship” is at the root of metaphors for aspects of human sociality without animal precedent—Christian brotherhoods, college sororities, father figures, and so on. But more importantly, it helps explain levels of human cooperation far higher than would be expected by primate standards, when people are tied together by the pseudo-kinship of everything from shared nationality or religion to shared intense partisanship for a sports team.

While the capacity to help a stranger based on an implicit sense of connectedness (the essence of pseudo-kinship) can make for a more benevolent world, it obviously can prompt something far from that as well. This is the militaristic world of “bands of brothers,” and considering everything from Masai warrior-class rituals to the basic training of the most technologically advanced armies, militaries excel at generating pseudo-kinship among their soldiers. This can produce circumstances that would be impossible among chimpanzees doing border patrols, namely a human combatant who shares more genes with his enemy than with the fellow fighter beside him (for example, a German-American fighting Nazis during World War II, alongside an Italian-American buddy).

The double-edge sword of pseudo-kinship is obvious in its potential to fuel co-operativity in groups of humans intent on killing other humans. But in addition, virtually of necessity, pseudo-kinship is accompanied by the human capacity to feel *less* related to someone than they really are, distancing an out-group individual to the point where they implicitly hardly feel human—pseudo-speciation (Berreby, 2008). History has no shortage of examples of the propagandistic pseudo-speciation that turns neighbors of a different religion into “a cancer growing in our culture,” that allows slaves to be classified as sub-human, or that leads to the characterization of undocumented people as an “infestation” (as has been done by an American president amid the current immigration debate). Pseudo-speciation is one domain that seems solely a single-edged sword.

## The Promise of Ridding us of Emotion, or Ridding us of Cerebration, Depending on Your Taste

Neuroscientists have long had to stave off dualism, often from the lay public, resisting simplistic notions of a dichotomy between brain and body, or mind and brain. One of the most durable of these dichotomies is the supposedly separate neurobiological domains of thought and emotion. As most clearly presented in neurologist Antonio Damasio's classic book, Descarte's Error (2005), this dichotomy is utterly false.

This can be seen on the neurobiological level. Much of the brain can be broadly (and, of course, falsely) dichotomized into two domains. One is the cortex, on the surface, standardly associated with the most abstract of thought. As might be expected, while occurring broadly among vertebrates, the cortex expanded greatly in relative size, evolutionarily, with the emergence of mammals; in turn, it is proportionately larger, successively, among primates, and apes and humans. Most interesting is the frontal cortex, the most recently evolved part of the human brain, a region that is bigger or arguably more complex in humans than in any other species. The frontal cortex has an array of functions that can be broadly summarized as “allowing us to do the right thing when that is the harder thing to do.” This includes gratification postponement, long-term planning, executive decision-making, regulation of emotions, and impulse control. The frontal cortex is, at first glance, the most cerebral part of the cerebrum.

In contrast is the “limbic system,” a circuit of brain regions, located beneath the surface of the cortex. In the early part of the Twentieth century, this region was termed the rhinencephalon (“nose brain”), as it received massive inputs in the rodent brain from the olfactory system; such labeling suggested that upwards of 40% of the brain was devoted to olfaction. Subsequent pioneering work showed that this region was, in fact, central to emotion, and the emotion-vs.-olfaction debate was resolved with the recognition that for a rodent, whose sensory world is overwhelmingly olfactory, emotion and olfactory social cues are inseparable. Much of the complex circuitry in the limbic system eventually converges on the hypothalamus at the base of the brain. The hypothalamus, in turn, regulates the release of hormones such as testosterone, estrogen, progesterone, and glucocorticoids, as well as regulating evolutionarily ancient autonomic function, such as blood pressure, heart rate, and body temperature.

This provided an easy dichotomy, where the limbic system's concerns were focused on the ancient base of the brain, providing the means by which emotion can alter the function of the body, while the cortex sat, both functionally and anatomically above it all. In this framework, the only communication between the cortex and the limbic system was inhibitory projections from the former to the latter, reining in imprudent emotional states. A classic example of this sort of regulation is shown in neuroimaging studies, where subjects are shown pictures of faces at nearly subliminal speeds. As a well-replicated finding, if the face rapidly displayed is that of an other-race individual, there is typically activation in a fraction of a second of the amygdala, an archetypal limbic structure. However, if the same experiment is run with exposure to each face on the order of seconds, the near instantaneous activation of the amygdala is then followed by frontal cortical activation, inhibiting amygdaloid activity. This is the effort seen in most people to suppress racist impulses (Kubota et al., 2012).

Thus, there is seemingly a stark anatomical and functional dichotomy between “emotion” and “cognition,” with interactions taking the form of the latter restraining the former. The falseness of this can first be seen anatomically, in that while there are the plentiful projections from the frontal cortex to limbic structures, there is an equivalent amount of signaling from the limbic system to the frontal cortex. Much of these projections converge on the ventromedial prefrontal cortex (vmPFC); the density of these inputs into the brain region that epitomizes the primacy and independence of cognition even lead to the heretical assertion by a renowned neuroanatomist that the vmPFC should be classified as part of the limbic system (Nauta, 1971). In other words, the limbic system influences the frontal cortex as much as the reverse.

This limbic influence can be appreciated with two examples. The first concerns memory. The remembering of facts—names, dates, images, and so on—seems profoundly cortical and abstract. Yet, the region of the brain most responsible for starting the process of remembering such information is the hippocampus, a structure sitting squarely in the limbic system. And in reflecting, this makes sense in a way that would be appreciated by Proust, namely that our most vivid memories are filtered through emotion. This determines what we remember (e.g., why we readily recall where we were on 9/11, but recall nothing of 9/10), and how it is remembered (e.g., the renowned inaccuracies of eyewitness accounts of aversive, dramatic events such as crimes). Moreover, our understanding of how memories, once consolidated, are recalled has been enriched by recent insights that emphasize this point as well. A simplistic picture of the process involves, in effect, taking a memory off its spot on a storage shelf, examining it as needed, and then returning it to the shelf as is. Instead, in a phenomenon called “reconsolidation,” the process of recalling, examining a memory alters it as a function of one's emotional state at the time; the memory placed back on the shelf can be different from the one first removed from it (Lee et al., 2017).

The second example of the limbic system shaping frontal cortical function concerns something whose consequences range from the everyday disruptive to the tragic, which is that during times of emotional arousal and stress, judgment and impulse control are greatly impaired. This will be discussed in the next section.

An understanding of limbic/frontal cortical interactions raises the temptation to therapeutically intervene, to alter the nature of those interactions. And what would constitute a helpful intervention differs dramatically depending on one's view of human nature and the roots of our societal ills. On one hand is the stance that the world would be less conflicted and violent if our behavior was increasingly dominated by the world of the frontal cortex—by thought, reflection, reasoning, emotional regulation and impulse control—a view that we are at our best, doing the right thing in a difficult circumstance, when we think our way to a moral stance. At the other end of the spectrum is the view that our best human moments are anchored in emotions, by feeling what it is like to walk a mile in another's shoes—by empathy, compassion and love—and that our finest moments arise from us feeling our way to a moral stance.

The double-edged quality to either of these propositions can be appreciated when considering the differing consequences of damage to either markedly “cerebral” or “emotional” parts of the brain. The punchline is that neither produces an ideal human. The first proposition would center on the dorsolateral prefrontal cortex (dlPFC), central to rational decision-making, the most recently evolved part of the frontal cortex, the nearest thing to the brain's “decider.” Selective damage to the dlPFC produces an organism (human or otherwise) whose behavior is dominated by limbic inputs funneled through the vmPFC. The individual is greatly impaired in planning or gratification postponement, acts impulsively in ways that can be sexually or aggressively disinhibited and highly damaging. To use a word with no basis in modern neuroscience beyond being metaphorical, this is an individual running entirely on id (Barbey et al., 2013).

A very different, but equally dysfunctional picture is produced in individuals with damage to the vmPFC, whose frontal cortex' deliberations occur in the absence of emotional input. Decisions are extremely difficult to make, because one lacks the “gut instinct” of limbic inputs that allows one to imagine how different outcomes to possible behaviors would feel (Damasio, 2005). There is a detached social inappropriateness; an individual with vmPFC damage, when meeting someone, might say, for example, “Hello, I see that you are considerably overweight” (and when castigated later by a mortified spouse, would respond with calm puzzlement, asking “What's wrong with that? It's true.”). Moreover, vmPFC damage produces someone who is pathologically utilitarian, who never hesitates to sacrifice one to save five, even if the one is a loved one (Bechara et al., 1994; Thomas et al., 2011).

Thus, the world would be unlikely to be a better place were we to function solely on thought, or emotion. The subtlety of the interactions of the two can be seen in a key issue in empathy research. There can be the temptation to view the capacity for empathy, to feel someone else's pain, as a virtue in and of itself, rather than merely one route by which to actually act with compassion. However, the transition from empathic feelings to compassionate actions is by no means guaranteed, and the disconnect is in the phenomenon of empathy fatigue—the danger of feeling someone's pain is that pain is painful, and if the vicarious pain is sufficiently severe, the pull becomes to turn away, to end one's own (empathic) pain. The more severe the emotional arousal when feeling empathy (for example, as measured by the increase in heart rate), the less likely a subject is to act pro-socially. As such, while emotions are at the core of empathy, emotional detachment can be central to acting on empathy (Eisenberg et al., 1994; Bloom, 2014).

The contrast between emotionally based and rationally based decision making has a strong time component to it. This distinction maps on to the dichotomy between System 1 and System 2 thinking, summarized by Daniel Kahneman in *Thinking, Fast and Slow* (2013), where the former is rapid, intuitive and emotional, whereas the latter is slow, rational and deliberative. The workings of both systems have double-edged qualities when it comes to moral decision-making and the potential for human conflict. As explored in Joshua Greene's *Moral Tribes: Emotion, Reason and the Gap Between Us and Them* (2014), social conflict can be grouped into two broad categories. The first concerns in-group members, where everyone agrees on the same general moral principles. In such cases, conflict is typically about an individual who, while theoretically adhering to those principles, is acting in a selfish manner filled with self-serving justification and rationalization. Greene terms this the problem of “Me vs. Us,” and likens this to the classic Tragedy of the Commons, where the problem is a free-rider in a setting of in-group cooperation. The second domain of conflict is “Us vs. Them,” between two groups with mutually contradictory beliefs, moral stances and senses of unalienable rights. The tragedy there is what Greene terms one of “commonsense morality,” where the problem is that each side has a passionately felt, common sense feeling about their own moral correctness, mistaking parochial intuition for logic and rationality.

Experimental economics work by David Rand and Greene (Rand et al., 2012) shows something important. Consider a “Me vs. Us” situation where an individual can either contribute their resources to a shared fund benefiting all or exploit the group's cooperativeness and selfishly keep the resources for themselves (while benefiting from everyone else's contributions to the group). Under such conditions, Greene and Rand show that the more rapidly a subject must decide what to do with their resources, the more pro-socially they act. The same is shown if they are prompted to think intuitively (by first being prompted by an interview to discuss a time where an intuitive decision on their part turned out to be better than a deliberative one would have been). In contrast, the more time subjects had to made a decision, or the more they were prompted to “carefully consider” their decision, the more selfishly they acted. In a world of in-group interactions, our rapid intuitions are pro-social and cooperative, and slow rational thought facilitates rationalizing why you should act selfishly and be an exception to an agreed-upon value.

The contrast with situations of Us vs. Them is obvious. Beginning with the amygdaloid response in most subjects in under 100 ms when seeing the face of an other-race individual, our most rapid, intuitive and emotional responses to out-group members tend toward fear, hatred, envy, and disgust. Pro-sociality toward an out-group member whose external symbols (e.g., dress, ornamentation) are alien, and whose beliefs are inaccessible or seem intuitively wrong, requires thought, perspective-taking, objectivity, and a willingness to perhaps uncomfortably examine oneself in a mirror.

## The Promise of Banishing Stress

Few things in physiology better constitute a double-edged sword than the stress response. With the onset of a stressful event, a wide range of adaptations are mobilized throughout the body, much of it built around the activation of the sympathetic nervous system, with its secretion of epinephrine (aka adrenalin) and norepinephrine, and the secretion from the adrenals of glucocorticoids, steroid hormones such as cortisol. If the stressful event is the sort of acute physical crisis that characterizes the vast majority of stress among other species (e.g., sprinting to evade a predator or sprinting after a meal), the stress response is absolutely central to surviving. But when that same stress response is chronically activated because of the psychosocial stress specialized in by humans, the result is an increased risk of cardiovascular, gastrointestinal, immunological and reproductive disorders (of note, throughout this section, “acute” stress and stress responses refers to time courses of seconds to an hour or two, whereas “chronic” refers to anything from a few hours to decades) (Sapolsky, 1995).

The dichotomy between the beneficial short-term effects of the stress response and the deleterious chronic effects extend to the brain as well. The original focus of research was on the hippocampus, where an acute stress response enhances oxygen and glucose delivery, and facilitates memory formation; in contrast, chronic activation of the stress response (primarily through the actions of glucocorticoids) impairs memory, and causes atrophy of hippocampal neurons (McEwen and Sapolsky, 1995). A different sort of dichotomy occurs in the amygdala, where the acute stress response is central to responding to an immediate threat, whereas chronic activation causes expansion of amygdaloid neurons and increased risk of anxiety disorders (Rodrigues et al., 2009).

In recent years, attention has shifted to the effects of stress on the frontal cortex, where there is a less clear-cut dichotomy concerning time course; instead, intense stress of any duration (or exposure to high levels of glucocorticoids) impairs frontal function. Working memory is disrupted, judgement is impaired, and coping strategies become perseverative and unresponsive to feedback (i.e., people cling to habitual problem solving, even if it is ineffective) (Young et al., 1999; Lyons et al., 2000; Dias-Ferreira et al., 2009; Schwabe and Wolf, 2010). This last effect involves poor cost/benefit analyses that bias in the direction of risk-taking, and is particularly pronounced in males (Starcke et al., 2008; Lighthall et al., 2009).

While intense stress decreases the activity of neurons in the frontal cortex, it activates the amygdala, allowing the latter to dominate the former (Roozendaal et al., 2004), thus increasing the odds of impulsive behavior. The results include increased levels of reactive aggression, (Mikics et al., 2007; Roelofs et al., 2007; Bertsch et al., 2011). These shifts are particularly striking when the intense stress or elevated glucocorticoid exposure is on the scale of weeks or more for laboratory rats. Under such circumstances, the changes in the amygdala and frontal cortex actually become structural, as amygdaloid neurons sprout new processes, enhancing the strength and reach of amygdaloid circuits and increasing the overall size of the structure (Vyas et al., 2002), while the opposite occurs in the frontal cortex (Radley and Morrison, 2006). These chronic stress effects are particularly damaging during development. Remarkably, having been raised under the stressful condition of a low socioeconomic status home produces five-year olds with elevated levels of glucocorticoids, and whose frontal cortices are likely to be thinner, less active, and less capable of regulating emotional behavior than average (Hackman et al., 2010; Sheridan et al., 2012).

Sustained stress or glucocorticoid exposure makes organisms less pro-social in additional ways. A number of studies have shown the rudiments of empathy in other species. Mice, for example, show a decrease in pain thresholds if in proximity to another mouse in pain (Langford et al., 2006); importantly, this pain resonance only occurs if the other mouse is a cage mate, as opposed to a stranger. The presence of a strange mouse is stressful, causing glucocorticoid secretion, and if the glucocorticoid release is blocked, a strange mouse's pain evokes a threshold shift as readily when the mouse is a cage mage (Martin et al., 2015). In other words, stress narrows a mouse's capacity for “empathy-like” behavior; moreover, the same glucocorticoid-dependent phenomenon occurs in humans (Martin et al., 2015). Stress narrows the concern for others in more ways; for example, exposing subjects to an experimental social stressor causes them to make more self-centered moral decisions (Starcke et al., 2011; Youssef et al., 2012).

Finally, stress also provokes aggression for the simple reason that aggression reduces stress. A number of things buffer the stress response during a psychological stressor; for a rat, this includes running on a running wheel, eating, or gnawing on wood in frustration. Amid this, one of the most effective buffers is for the rat to be able to bite another individual (Levine et al., 1988). Such displacement aggression is a reliable stress reducer across the animal kingdom. Among baboons, for example, about half of aggression is displacement aggression, and for the same dominance rank, the more a baboon tends to displace aggression after losing a fight, the lower his glucocorticoid levels (Sapolsky and Ray, 1989). The increase in rates of spousal and child abuse during times of economic stress is but one of the many examples of stress-induced displacement aggression in humans.

Thus, stress makes organisms fearful, more egoistic and less empathic, less likely to think clearly, assess risks accurately, incorporate new data, or to restrain impulses. In a world filled with the violent consequences of poor judgment and poor impulse control, it is tempting to imagine a better world thanks to neurobiological interventions that block the stress response. This is not possible from a mundane standpoint of health—while eliminating the stress response might decrease the risks of a stress-related degenerative disorder such as atherosclerosis, it would kill the individual the first time they ran for a bus; the stress response is essential for life when an organism, human or otherwise, is dealing with an acute physical demand. Eliminating the stress response is also undesirable for the important reason that moderate and transient stress is pleasurable, something we seek out—stress under such circumstances is what we call stimulation.

For the purposes of this paper, the strongest reason not to eliminate the stress-response is because of the double-edged nature of its effects on the brain and behavior. The adverse effects of stress on decision-making and impulse control are value-free, are “adverse” only in the instrumental and neurobiological sense. In the middle of a stressful crisis, an EMT may act in perseverative and impulsive ways, making her ineffectual at saving lives; blunting the effects of stress and glucocorticoids on the brain at that time is beneficial. But likewise, in the middle of a stressful crisis, a sociopathic warlord may act in perseverative and impulsive ways, making him ineffectual at ethnically cleansing a village. Eliminating the stress response in that circumstance would be anything but beneficial.

## Conclusions

This review has considered a variety of areas where advances in behavioral neuroscience relevant to human conflict have generated enormous excitement. In each case, seemingly straightforward interventions prompted by these findings (such as fostering neuroplasticity, or using oxytocin to promote pro-sociality) instead come with significant double-edged swords. The enthusiasm with which some of these findings have garnered translational, or even therapeutic interest has to make one worry about the hubristic interventions in the past. Behavioral neuroscience should long be shadowed by frontal lobotomies, amygdalectomies for supposedly intractably violent individuals, chemical or surgical castration for sex offenders, and other horrors of our disciplinary past (Valenstein, 2010).

But the purpose of this review was not just to urge caution about new findings. These double-edged swords not only reflect the complexity of the topics, but the particular nature of the complexity. What is shown is that a hormone can have diametrically opposite effects on behavior in different settings, that the workings of the same small brain region can give rise to either pro- and anti-social behavior. The biology of sociality repeatedly shows contingent effects with profound dependencies on context. This particular type of complexity is central to the most puzzling features of our behavior—we are simultaneously the most violently destructive species on this planet and the most altruistic and empathic; we as individuals can be monstrously damaging in one setting and profoundly caring in another; the same behavior can constitute humans at their best in the eyes of some and humans at their worst for others. It will be critical to understand these seeming contradictions.

## Author Contributions

The author confirms being the sole contributor of this work and has approved it for publication.

### Conflict of Interest Statement

The author declares that the research was conducted in the absence of any commercial or financial relationships that could be construed as a potential conflict of interest.
